# Premilling treatments effects on nutritional composition, antinutritional factors, and in vitro mineral bioavailability of the improved Assosa I sorghum variety (*Sorghum bicolor* L.)

**DOI:** 10.1002/fsn3.2155

**Published:** 2021-02-03

**Authors:** Ebisa Olika Keyata, Yetenayet B. Tola, Geremew Bultosa, Sirawdink Fikreyesus Forsido

**Affiliations:** ^1^ Department of Food Science and Nutrition Wollega University Shambu Ethiopia; ^2^ Department of Post‐Harvest Management Jimma University College of Agriculture and Veterinary Medicine Jimma Ethiopia; ^3^ Department of Food Science and Technology Botswana University of Agriculture and Natural Resources Gaborone Botswana

**Keywords:** antinutrients, bioavailability, malting, minerals, proximate, soaking, sorghum

## Abstract

Sorghum (*Sorghum bicolor* L.) is among the staple cereal crops in different parts of Ethiopia. However, the presence of antinutritional factors restricts the digestion of proteins and bioavailability different minerals. Therefore, this study investigates the premilling treatments effects on nutritional composition, antinutritional factors, and in vitro mineral bioavailability of the improved Assosa I sorghum variety grown in Benishangul‐Gumuz Region, Ethiopia. The experiment was conducted in a completely randomized design with single factor of premilling treatments (control, washing, soaking, and malting). Among evaluated premilling treatments, malting showed significant (*p* < .05) increase in terms of crude fiber, utilizable carbohydrate, gross energy, and sodium contents. As compared to the raw sorghum, premilling treatments reduced antinutritional contents from 55.81 to 27.4 mg/100 g for tannin, 156.15 to 70.50 mg/100 g for phytates, and 29.9 to 3.22 mg/100 g for oxalate. The premilling techniques also significantly (*p* < .05) improved in vitro mineral bioavailability as compared to unprocessed sorghum grains. Among the premilling treatments, malting showed significant difference (*p* < .05) in terms of reduction of tannins, phytates, and oxalate contents with relatively higher mineral bioavailability. In order to enhance the food and nutritional value of sorghum particularly for children and lactating mothers, it is recommended to germinated the grains. Flour from germinated grain also can be used in combination with other nutrient‐dense foods to formulate healthy diets for children and maternal nutrition.

## Graphical abstract

Malting significantly decreases antinutritional factors with high improvement of in vitro mineral bioavailability. Soaking increases mineral contents of sorghum due to adsorption of minerals from tap water. All premilling treatments such as washing, soaking, and malting reduce antinutritional contents with improvement of in vitro mineral bioavailability

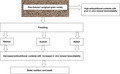

## 1 INTRODUCTION

Sorghum (*Sorghum bicolor* L.) is the sixth most planted crop in the world, and the second cereal grain in Africa, which is used as human staple food grain in many semiarid and tropical areas of the world (Zhao et al., 2019). Notably in sub‐Saharan Africa and Asian continent, approximately 500 million of the poorest and most food‐insecure people rely on sorghum for their protein and energy requirements (Gebreyes, 2017). According to Food and Agricultural Organization of the United Nations, about 76.3% of Ethiopian populations rely on consumption of sorghum (FAO, 2017). The grain is the third important staple cereal crop after maize and teff in Ethiopia (CSA, 2019), which is processed into flour and used in a variety of staple foods for the general public and children under age of five (Tasie & Gebreyes, 2020).

Regarding nutritional point of view, sorghum is a good source of carbohydrate (55.2%–72.2%), protein (8.6%–18.9%), ash (1.1%–2.4%), oil (1.7%– 4.9%), and fiber (9.3%–25.2%) (Queiroz et al., 2015). Gerrano et al. (2016) also reported that sorghum flour contained (in mg/kg) 195.0 – 477.0 Ca, 950.0 – 2,146.9 K, 14.5–58.6 Na, 28.8–55.1 Fe, and 12.0–23.0 Zn. Despite to this, sorghum grains contain different antinutritional factors like tannins, phytic acids, and oxalate in relatively higher concentration as compared to other cereal crops (Ojha et al., 2018). The antinutritional factors have a negative impact in human nutrition by hindering bioavailability through binding of important minerals (Fe, Ca, and Zn) and digestibility of proteins that interferes on growth, reproduction, and health of the general public and in particular children under ages of five (Popova & Mihaylova, 2019).

In Ethiopia, sorghum is consumed without doing any efforts to minimize the antinutritional contents. However, there are premilling processing techniques that can help to reduce the high concentration of antinutritional factors to improve bioavailability of minerals and digestion of proteins (Samtiya et al., 2020; Tamilselvan & Kushwaha, 2020) and organoleptic properties (Oghbaei & Prakash, 2016).

Assosa I, a sorghum variety is a highly productive, stress tolerant improved variety released by Ethiopian Institute of Agricultural research. Even though staple food crop widely distributed and produced in the region, the antinutritional contents and means to minimize the contents were not released as part of the technology package for the users. Therefore, this study was initiated to investigate the impact of premilling treatments to minimize the concentration of antinutritional factors to acceptable level for better nutrition and health of the community.

## 2 MATERIALS AND METHODS

### 2.1 Experimental material

Sorghum selected for this study was grown in Benishangul‐Gumuz Region (BGR) in the western part of Ethiopia which is one of the dominant sorghum producing areas in Ethiopia, where the crop is used as staple food for the majority of the people in the region (CSA, 2019). Based on this context, Assosa I sorghum variety (14 kg) was collected from Assosa Agricultural Research Centre (AARC), Ethiopia. The grains were cleaned manually by removing any dockages (foreign material, damaged and broken seeds, and shriveled and insect‐attacked grains).

### 2.2 Experimental design

The experimental design used was a completely randomized design (CRD) with single factor (processing technique) of four levels (control = raw, washed, soaked, and malted) replicated three times (12 experimental units).

### 2.3 Premilling techniques

**FIGURE 1:**
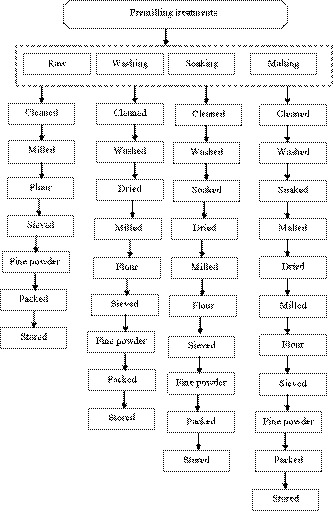
Premilling treatments effects on the improved Assosa I sorghum grain variety

The control unprocessed sorghum grains were after cleaning directly milled into flour. For the washing, the cleaned sorghum grains were washed three times with tap water and dried. For the soaking, cleaned sorghum grain samples were washed three times and steeped in sorghum: tap water ratio (1:5, w/v) at 25°C for 6h in a steeping vessel containing 0.2% NaOH solution (Bekele et al., 2012). At the end of 6 hr, the vessel drained and then refilled with fresh tap water at room temperature. The water then drained, and refilled every 3 hr for the next 18 hr, with a 1‐hr air rest between each refill. Then, the steeped grains were dried at 50°C for 24 hr. For the treatment of malted sorghum grains, steeping procedure was done as described for soaking treatment. The steeped sorghum grains were then germinated in a germination vessel at room temperature and approximately at 95% relative humidity for 41 hr (after preliminary test). The germinating grains were turned occasionally to avoid meshing of the roots and shoots. At the end of each germination treatment, the samples were removed from the vessel and then dried in an oven (DHG‐9203A, Shanghai, China) at 50°C for 24 hr. All the processed sorghum grain samples were milled separately into the flour using laboratory mill (RRH‐200) to pass through 0.5 mm sieve size (AACC, 2000). The flours were packed in heavy‐duty polyethylene bags wrapped using aluminum foil and stored at 4°C until used for analysis. The premilling treatments flowchart is as given in Figure 1.

### 2.4 Proximate composition and energy content

The moisture content of the samples was determined by oven (DHG 9,203, Shanghai, China) drying method (105°C for 6 hr) by taking about 3 g sample (dried sample powder) as described in the AOAC (2000) method 925.10. Crude protein content was determined by Kjeldahl method (VELP. Scientifica UDK 159, Automatic distillation & titration system 230V) of nitrogen content analysis after digestion of about 1.0 g of sample as described in AOAC (2000) method 920.87. The crude fat content was determined by taking about 1.5 g of sample by Soxhlet extraction (SZC‐C, Shanghai Qianjian, China) method using petroleum ether as a solvent (AOAC, 2000, method, 920.39). The crude fiber content was determined following AOAC (2000) method 962.09 after sequential digestion with 1.25% H_2_SO_4_ and 28% KOH, screening through 75 micron, drying and ignition in a muffle furnace (Sx2‐4–10, Zhejiang, China) to subtract ash from crude fiber. The total ash content was determined gravimetrically after carbonization of about 2.0 g sample on a blue flame of Bunsen burner followed ignition at 550°C until ashing complete (AOAC, 2000, method 923.03). Utilizable carbohydrate content (UCC) was calculated by the difference method (FAO, 1998), and gross energy content was calculated by Atwater's conversion factors (FAO, 2002).

### 2.5 Mineral content

The mineral content of the samples was determined by atomic absorption spectrophotometer (AAS) (Agilent FS240 AA, USA) following AOAC (2000) method 985.35 after carbonization on a heating plate and dry ashing of about 3.0 g of samples in a muffle furnace (Sx2‐4–10, Zhejiang, China) at 550°C until ashing was completed. The white ash was dissolved using 5 ml of 6N HCl, dried on a hot plate followed by addition of seven ml of 3N HCl heating on a hot plate, and then, finally the solution was diluted to the mark (50 ml) with deionized water. The Ca, Fe, and Zn contents were determined by AAS using air–acetylene as a source of flame energy for atomization. The absorbance for Fe was measured at 248.3 nm, and the Fe content was estimated from a standard calibration curve (0, 0.5, 1, 2, 3, and 4 µg/ml) prepared from analytical grade iron wire. The absorbance for Zn was measured at 213.9 nm, and the Zn content was estimated from a standard calibration curve (0, 0.125, 0.250, 0.50, 0.75, and 1 µg/ml) prepared from ZnO. The absorbance of Ca was measured at 422.7 nm after addition of 2.5 ml of LaCl_3_ to the sample solution. The Ca content was then estimated from the standard solution (0, 0.5, 1, 1.5, 2, and 2.5 µg/ml) prepared from CaCO_3_. The Na and K contents were determined using flame photometer (ELICO CL 378, India) by measuring their emission at 589 and 767 nm, respectively. The Na content was estimated from standard solution (0, 0.5, 1, 1.5, 2, and 2.5 µg/ml) prepared from NaCl. The K content was estimated from a standard solution (0, 2, 4, 6, 8, 10, and 12 µg/ml) prepared from KCl.

The mineral elements content was calculated using the Equation 1:

(1) Element(mg/100g)=[(μg/mL)×DF]/[(samplemass,db)×10]

where DF = dilution factor (50 ml for Ca, Fe, Zn, K, and Na), and db is sample mass on dry matter basis.

### 2.6 Antinutritional factors

#### 2.6.1 Determination of condensed tannins content

The condensed tannins content was determined according to the method described by Maxson and Rooney (1972). About 1.000g of the sample was weighed and mixed with 10 ml of 1% HCl solution in methanol in a screw cap test tube. Then, the tube was shaken for 24 hr at room temperature on a mechanical shaker (Hy‐2(C), Shanghai, China). The solution was centrifuged (sigma 2‐16KC, UK) at 1,000 rpm for 5 min. One ml of supernatant was transferred to another test tube and mixed with 5 ml of vanillin–HCl reagent (prepared by combining equal volume of 8% concentrated HCl in methanol and 4% vanillin in methanol). The D (+)‐catechin was used as a standard for condensed tannins content determination. A 40 mg of D (+)‐catechin was weighed and dissolved in 1,000 ml of 1% HCl solution in methanol, which was used as stock solution from which a series of standard solutions (0, 12, 24, 36, 48, and 60 μg /ml) were prepared by mixing with 5 ml 1% HCl in methanol. The absorbance of samples and the standard solutions were measured at 500nm using UV‐VIS Spectrophotometer (JASCO V‐630, Shimadzu Corporation, Tokyo, Japan) after 20 min. The condensed tannins content was determined from standard curve of catechin, and result was expressed as mg/100g.

#### 2.6.2 Determination of phytate content

Phytate content was determined by the method described by Vaintraub and Lapteva (1988). About 0.100 g of sample was extracted with 10 ml of 2.4% HCl in a mechanical shaker (Hy‐2(C), Shanghai, China) for 1 hr at a room temperature. The extract was centrifuged (Sigma 2‐16KC, UK) at 3,000 rpm for 30 min. The clear supernatant was used for phytate estimation. One ml of wade reagent (containing 0.03% solution of FeCl_3_.6H_2_O and 0.3% of sulfosalicylic acid in water) was added to 3 ml of the sample solution (supernatant), and the mixture was mixed on a vortex mixer for 5 s. The absorbance of the sample solutions was measured at 500 nm using UV‐VIS spectrophotometer (JASCO V‐630, Shimadzu Corporation, Tokyo, Japan).). A series of standard solutions from sodium salt of phytic acid were prepared to contain 0.0, 4.5, 9.0, 18.0, 27.0, and 36.0 μg/ml of phytic acid (analytical grade sodium phytate) in 0.2N HCl. One ml of the wade reagent was added to each test tube, and the solution was mixed on a Vortex mixer for 5 s. The mixture was centrifuged for 10 min, and the absorbance of the sample and standard was measured at 500 nm by using deionized water as a blank. The phytate content was determined from standard curve of sodium salt of phytic acid, and result was reported in mg /100g.

#### 2.6.3 Determination of oxalate content

The oxalate contents were determined using the method of AOAC (2000) method 974.24. About 2g samples were suspended in 190 ml of distilled water contained in a 250 ml volumetric flask; 10 ml of 6M HCl was added, and the suspension was digested at 100°C for 1 hr, followed by cooling and then made up to 250 ml before filtration. A 125 ml of the filtrate was measured into a beaker, and four drops of methyl red indicator were added, followed by the addition of concentrated NH_4_OH solution (dropwise) until the test solution changed from its salmon pink color to a faint yellow color (pH 4–4.5). Each portion was then heated to 90°C, cooled, and filtered to remove precipitate containing ferrous ion. The filtrate was again heated to 90°C, and 10 ml of 5% CaCl_2_ solution was mixed while being stirred constantly. After heating, it was cooled and left overnight at 5°C. The solution was then centrifuged (Sigma 2‐16KC, UK) at a speed of 2,500 rpm for 5 min. The supernatant was decanted, and the precipitate was completely dissolved in 10 ml of 20% (v/v) H_2_SO_4_, solution. The total filtrate resulting from digestion of 2g of the sample was made up to 300 ml. Aliquots of 125 ml of the filtrate was heated until near boiling and then titrated with standard 0.05M KMnO_4_ solution to a faint pink color which persisted for 30s. The calcium oxalate content was expressed as calcium oxalate equivalent and calculated using the formula:

$$\text{Oxalate}\left(\frac{\text{mg}}{100\;\text{mg}}\right)=\frac{T\times V_{\text{me}}\times \text{DF}\times \text{10}\;S}{\text{ME}\times \text{MF}}$$

where T is the titer of KMnO_4_, (ml), V_me_ is the volume ‐mass equivalent (i.e., 1 cm^3^ of 0.05 M KMnO_4_ solution is equivalent to 0.00225 g anhydrous oxalic acid), DF is the dilution factor (2.4), where the total volume of filtrate (300 ml) divided by aliquot used (125 ml) for titration, ME is the molar equivalent of KMnO_4_ in oxalate (KMnO_4_ redox reaction), and MF is the mass of flour used.

### 2.7 Molar ratios and bioavailability of minerals

The molar ratio of phytate to minerals (Ca, Zn, and Fe) was calculated by dividing the mole of phytate (mass of phytate/660 g/mol) to the mole of minerals (mass of Ca = 40 g/mol; mass of Zn = 65 g/ mol; mass of Fe = 56 g/mol) (FAO, 2018). Also, the oxalate to calcium molar ratio was calculated by dividing the mole of oxalate (88 g/mol) to calcium mole. The molar ratios found were also compared with the critical toxicity values described in WHO/FAO (2004).

### 2.8 Statistical analysis

The triplicate data were subjected to analysis of variance (ANOVA). All the statistical analyses were performed using SAS version 9.3, and significance difference was considered at *p* ≤ .05. *Fisher's* least significant difference (LSD*)* was used for mean comparison tests to identify significant differences among means (*p* ≤ .05). The results were expressed as mean ± standard deviation.

## 3 RESULTS AND DISCUSSION

### 3.1 Proximate composition and energy contents of Assosa I improved sorghum variety

The processing methods had a significant (*p* < .05) effect on the moisture content of the sorghum flour samples (Table 1). The result showed that the highest moisture content (8.70%) was recorded for directly milled unprocessed sorghum grain flour, and the lowest (5.21%) was from malted sorghum grains flour. This might be in part due to drying of treated samples for extended drying time but not the case for raw sample. On sorghum grains malting, there is grains constituent modification under controlled conditions, starch granules are modified in part hydrolyzed, and this resulted into reduced water‐binding capacity and that is why low moisture content was recorded in the malted sorghum grains. Almost similar moisture content for raw, soaked and malted, sorghum grain flours (8.38 to 8.76, 6.07 to 6.56, and 5.33 to 6.37%, respectively) were reported by Afify et al. (2012).

**TABLE 1:** Premilling treatments effects (washing, soaking, and malting) on proximate composition (% db) and energy (kcal/100 g db) contents of Assosa I improved sorghum grain variety

Premilling	MC	PC	FC	FiC	AC	UCC	Energy
Control	8.7 ± 0.6^a^	9.55 ± 0.03^a^	3.68 ± 0.02^a^	2.96 ± 0.02^b^	1.60 ± 0.03^a^	82.2 ± 0.1^d^	400.2 ± 0.3^c^
Washed	6.4 ± 0.6^c^	8.0 ± 0.1^c^	3.7 ± 0.1^a^	2.79 ± 0.03^c^	1.52 ± 0.04^b^	83.98 ± 0.04^c^	401.4 ± 0.3^b^
Soaked	6.9 ± 0.5^b^	7.9 ± 0.3^c^	3.51 ± 0.05^b^	1.67 ± 0.02^d^	1.01 ± 0.02^c^	85.9 ± 0.2^a^	406.8 ± 0.2^a^
Malted	5.2 ± 0.5^d^	8.44 ± 0.03^b^	3.3 ± 0.1^c^	3.00 ± 0.03^a^	1.06 ± 0.02^c^	84.2 ± 0.2^b^	400.3 ± 0.6^c^
CV	0.81	1.64	2.41	0.84	2.00	0.10	0.11
LSD	0.11	0.28	0.17	0.044	0.05	0.16	0.85

Note

Control = unprocessed sorghum grains. Means with different letters across a column are significantly different.

Abbreviations: AC, ash content; FC, fat content; FiC, fibre content; MC, moisture content; PC, protein content; UCC, utilizable carbohydrate content.

There was a significant (*p* < .05) difference in the protein content between control (unprocessed) and processed samples. However, there was no significant (*p* > .05) effect on the protein content between the washed and soaked sorghum grains. Among premilling processing methods, higher protein content was observed in malted sorghum grains, but was less than the protein content in the raw sorghum grains (Table 1). This might be because of the loss of water‐soluble nitrogenous compounds during rinsing, soaking of grains and utilization of protein for the growth and development of the embryo on germination of sorghum grains (Nour et al., 2015). However, the protein content of the malted sorghum grains found in this study was higher than the values reported for malted sorghum grains elsewhere (6.90%) (Tamilselvan & Kushwaha, 2020), which could be possibly to the difference in the genetic makeup and agronomic practices. The protein content obtained from unprocessed sorghum grains was similar to the value reported for sorghum grain varieties grown in Ethiopia (8.20 to 16.48%) (Tasie & Gebreyes, 2020).

The study showed that there was no significant difference (*p* > .05) in the crude fat contents between raw and washed sorghum grains. However, soaked and malted sorghum grains had significantly (*p* < .05) lower crude fat content compared to the control sample. Different authors also indicated that the negative impact of malting process in terms of reducing the fat content of sorghum, pearl millet and maize grains (Derbew & Moges, 2017; Inyang & Zakari, 2008; and Kikafunda et al., 2006). The decrease in the fat content could be due to an increase in the activity of lipolytic enzymes during germination, which hydrolyzed the fats into fatty acid and glycerol (Onweluzo & Nwabugwu, 2009).

The fiber content was significantly (*p* < .05) affected by the processing methods. The value for raw sorghum grains (2.96%) was reduced to 1.67% and 2.79% as a result of washing and soaking, respectively (Table 1). A similar reduction of fiber content in the soaked and increment of fiber content in the malted sorghum grains was also reported from Egypt (Afify et al., 2012). The decline in the fiber content of soaked sorghum might be due to increase in the activity of β‐glucanase enzyme which could reduce the fiber contents of sorghum on the soaking (Mathur & Choudhry, 2009) while the increase upon malting, might be attributed to the synthesis of structural carbohydrates, such as cellulose and hemicelluloses during germination (Pandey & Awasthi, 2015). The increase in the fiber content is desirable for people with prevention of obesity, cardiovascular disease, type 2 diabetes, and large intestine cancer (Champ et al., 2003), but might not desired to formulate complementary foods for children.

Total ash content shows that premilling processing methods had significantly (*p* < .05) decreased the ash content. Similar reduction of the ash content was reported by Gernah et al. (2011) on malting of sorghum grains. The ash content of soaked and malted sorghum grains was significantly (*p* < .05) decreased when compared to unprocessed sorghum grains, but it was not significantly (*p* > .05) different from each other. Similar reduction of ash contents during soaking and malting also reported by Keyata et al. (2018). The reduction of ash content during soaking and malting might be due to leaching out of some minerals. The decrease in the ash content of malted sorghum can be due to the consumption of minerals during the growth of the germ (Mubarak, 2005).

Table 1 shows the processed and unprocessed sorghum grains had a significant (*p* < .05) effect on the utilizable carbohydrate content (UCC). The higher UCC (85.91%) was recorded in the soaked sorghum grains followed by malted grains (84.18%) while the lower UCC (82.21%) was observed in the raw sorghum grains followed by washed sorghum grains (83.98%). The result indicated that processing methods have increased the content of utilizable carbohydrate of sorghum grains flour. This could be attributed to the reduction of the moisture, protein, ash, fat (particularly in soaked and malted sorghum), and fiber (specifically in washed and soaked) contents, since UCC was calculated by differences.

The processing methods had significant (*p* < .05) effect on the gross energy content. The highest energy content (406.83 kcal/100g) was noted for soaked sorghum grains followed by the washed sorghum grains (401.39 kcal/100g). However, the lowest gross energy content (400.19 kcal/100g) was recorded in unprocessed sorghum grains sample followed by malted sorghum grains (400.28 kcal/100g), but with no significant difference (*p* > .05) between them. This might be attributed to the relatively low‐fat content obtained in malted sorghum grains and less carbohydrate content recorded in the unprocessed sorghum grains sample.

### 3.2 Mineral content

Minerals play a vital role in the maintenance of human health. Cereal grains are rich in minerals, but the bioavailability of these minerals is usually low due to the presence of antinutritional factors (Nadeem et al., 2010). Processing methods before milling showed significant (*p* < .05) effect on the mineral content of samples (Table 2). The results showed an increase in the mineral content for soaked samples but a decrease for malted grains. An increase from the soaked grain samples might be in part contributed to the adsorption of minerals from tap water during soaking time (Claver et al., 2011). Furthermore, more minerals could be released during soaking due to release of bounded minerals with phytate because of solubility of phytate in water (Nour et al., 2015; Vashishth et al., 2017). Claver et al. (2011) reported that sorghum soaked for 6 to 24 hr had significantly increased Na, Ca, and Zn contents. Similar result in terms of increments of Ca, K, and Na contents during soaking of wheat and oat grains was also reported (Samia et al., 2013). The decrease in the mineral contents during malting could be due to the use of the minerals in the process of germination by the growing embryo (Udeh et al., 2018). For instance, the decrease in the zinc content during the malting process could be due to the use of zinc in cell reproduction and tissue growth (Scherz & Kirchhoff, 2006). Omoikhoje and Obasoyo (2018) indicated a decrease in K, Na, Fe, and Zn contents during germination of sorghum grains and similar decrease in Ca, K, Fe, and Zn contents in malted sorghum grains were reported (Claver et al., 2011; Samia et al., 2013), whereas the increase in the sodium content of malted sorghum grains was reported by Claver et al. (2011). The increment of sodium during malting probably was a result of the conversion of the insoluble reserve foods enzymes in the grains (Rooney & Serna‐Saldivar, 1991).

**TABLE 2:** Premilling treatments effects (washing, soaking, and malting) on mineral (mg/100 g db) contents of Assosa I improved sorghum grain variety

Premilling	Sodium	Potassium	Calcium	Iron	Zinc
Control	29.5 ± 2.2^c^	333.4 ± 6.4^c^	34.02 ± 0.34^b^	3.0 ± 0.1^c^	0.93 ± 0.03^ba^
Washed	31.9 ± 1.9^c^	454.04 ± 0.40^a^	34.1 ± 0.8^b^	4.84 ± 0.09^b^	0.89 ± 0.01^c^
Soaked	39.4 ± 1.9^b^	399.2 ± 1.3^b^	35.3 ± 0.1^a^	5.13 ± 0.06^a^	0.97 ± 0.02^a^
Malted	47.2 ± 1.0^a^	323.9 ± 2.4^d^	33.0 ± 0.2^c^	2.97 ± 0.1^c^	0.91 ± 0.02^bc^
CV	4.62	0.920	1.32	2.69	2.29
LSD	3.42	6.94	0.90	0.21	0.04

Note

Control = unprocessed sorghum grains. Means with different letters across a column are significantly different.

### 3.3 Antinutritional factors content

The impact of processing methods on antinutritional contents of tannins, phytates, and oxalate are given in Table 3.

**TABLE 3:** Premilling treatments effects (washing, soaking, and malting) on antinutritional contents (mg/100 g, db) of Assosa I improved sorghum grain variety

Premilling	Tannin	Reduction (%)	Phytate	Reduction (%)	Oxalate	Reduction (%)
Control	55.81 ± 0.75^a^	‐	156.15 ± 0.24^a^	‐	29.9 ± 0.8^a^	‐
Washed	52.2 ± 1.5^b^	6.42	131.8 ± 0.6^b^	15.57	17.85 ± 0.46^b^	40.30
Soaked	36.80 ± 1.05^c^	34.06	88.0 ± 0.7^c^	43.66	5.8 ± 0.2^c^	80.47
Malted	27.4 ± 0.5^d^	49.04	70.5 ± 1.1^d^	54.87	3.22 ± 0.02^d^	89.23
CV	2.66		0.72		2.57	
LSD	2.28		1.61		0.73	

Note

Control = unprocessed sorghum grains. Means with different letters across a column are significantly different.

Sorghum grains bear antinutritional factors, particularly condensed tannins which may form stable complexes with protein, metal ions, and other macromolecules like polysaccharides and can result in reduction of the digestibility of proteins and limit availability of the nutrients in the gut (Selle et al., 2010). Therefore, application of different premilling processing methods could have potential to reduce the condensed tannins content. Results of this study show that processing methods had significantly (*p* < .05) decreased the condensed tannin contents. The condensed tannin contents reduced significantly after malting, soaking, and washing by 49.04, 34.06, and 6.42%, respectively. This finding revealed that malting process had the highest reductions of condensed tannin contents followed by soaking of sorghum grains. The decrease in the condensed tannin during malting was also reported in sorghum in other study (Ojha et al., 2018) and in other cereal grains (Forsido et al., 2020). This might be due to water solubility of condensed tannins mainly concentrated in the seed coat of grains, for which can be easily leached out during washing and soaking (Ogbonna et al., 2012).

Phytic acid is mostly concentrated in the bran (aleurone layer) of grains and germ, which may lower bioavailability of minerals and digestibility of proteins and carbohydrates, by inhibiting the normal activity of digestive enzymes like pepsin, trypsin, and amylase (Kumar et al., 2010). In this context, premilling treatments applied in this study had significantly (*p* < .05) reduced the phytic acid contents in the sorghum grains. The phytic acid content was reduced by 15.6%, 43.7%, and 54.9% after washing, soaking, and malting treatments, respectively. The activation of endogenous phytase enzymes in the grain during malting time could hydrolyze phytates structure and resulted in a decrease in the concentration of phytates. A similar reduction of phytic acid content was observed during soaking and germination of chickpea (Olika et al., 2019), malted sorghum grains (Ojha et al., 2018), malted teff, barley, and oats grains (Forsido et al., 2020).

Oxalic acid and its salts can have deleterious effects on human nutrition and health, particularly by decreasing calcium absorption and aiding the formation of kidney stones (Gemede, 2020). The premilling treatment methods showed significant (*p* < .05) impact on the reduction of oxalate content in the sorghum grains. The oxalate content of unprocessed sorghum grains was 29.9 mg/100 g but the concentration was reduced by 40.30%, 80.47%, and 89.23% after washing three times, soaking for 18 hr, and malting for 41 hr, respectively. The result showed that sorghum malting significantly contributed to the decrease of oxalate concentration in the grain. This might be due to leaching of oxalate compound in water during combined processing technique such as washing, soaking, and malting by itself. Similar results were reported for reduction of oxalate content in soaked and malted barley grains (Brudzyński & Salamon, 2011). However, the oxalate content obtained from both unprocessed and processed sorghum grains in this study was below the recommended daily intake range for human consumption (maximum tolerated level of 50 mg/100 g) (Massey et al., 2001).

### 3.4 Molar ratios and bioavailability of minerals

Total and bioavailable mineral content is negatively influenced by antinutritional factors such as phytate, tannin, and oxalate. In order to increase the bioavailability of mineral, premilling treatments and processing have a positive impact through reducing the concentration of mineral inhibitors and favorably altering food components into complex ligands for metal ions (Rousseau et al., 2020). The calculated values of the molar ratios compared with the reported critical toxicity values are given in Table 4.

**TABLE 4:** Premilling treatments effects (washing, soaking, and malting) on molar ratio between antinutritional and mineral content of Assosa I improved sorghum grain variety

Premilling	Ph:Ca	Ph:Fe	Ph:Zn	Ox:Ca	Ph*Ca:Zn
Control	0.28 ± 0.004^a^	4.5 ± 0.2^a^	16.5 ± 0.4^a^	0.40 ± 0.007^a^	14.0 ± 0.5^a^
Washed	0.24 ± 0.005^b^	2.31 ± 0.03^b^	14.6 ± 0.1^b^	0.24 ± 0.001^b^	12.4 ± 0.2^b^
Soaked	0.15 ± 0.002^c^	1.46 ± 0.01^d^	8.9 ± 0.3^c^	0.075 ± 0.003^c^	7.9 ± 0.2^c^
Malted	0.13 ± 0.003^d^	2.02 ± 0.04^c^	7.7 ± 0.2^d^	0.044 ± 0.001^d^	6.32 ± 0.15^d^
Favorable ratio	<0.17	<1 (pref. < 0.4)	<15	<1	<200
CV	1.69	3.91	2.57	1.59	2.93
LSD	0.01	0.20	0.61	0.01	0.60

Note

Control = unprocessed sorghum grains, Ph:Ca: phytate:calcium, Ph:Fe: phytate: iron, Ph:Zn: phytate: zinc, Ox:Ca, oxalate: calcium, Ph*Ca:Zn, phytate *calcium: zinc. Means with different letters across a column are significantly different.

Premilling treatments significantly (*p* < .05) improved the bioavailability of calcium content in washed (0.24), soaked (0.15), and malted sorghum grains (0.13) as compared to the control sorghum grain sample (0.28). The phytate:Ca molar ratio less than 0.17 is an indicator of Ca bioavailability (Castro‐Alba et al., 2019). The result showed that absorption of calcium in soaked and malted sorghum is most probably not adversely affected by phytate.

The phytate:Fe molar ratio has to be lower than 1.0 and preferably lower than 0.4 for favorable iron absorption (Hurrell, 2004). In this concern, all premilling treatment methods had improved iron bioavailability when compared to the control sorghum grain sample. However, all treated sorghum grain samples have above a critical value (less than 1.0) of phytate:Fe molar ratio reported by Hurell (2004). The poor iron bioavailability in the raw and premilling treatments of sorghum grains might be due to the reported higher phytate content and insufficient phytic acid degradation (Hailu & Addis, 2016).

The phytate to Zn ratio of less than 10 shows adequate availability of Zn, and problems are encountered when the value is greater than 15 (Morris & Ellis, 1989). In this regard, processing methods had significantly (*p* < .05) increased zinc bioavailability in sorghum. Among the processing methods applied, sorghum grain malting had the highest zinc bioavailability followed by soaked sorghum grains while washed sorghum grains had the lowest.

Frontela et al. (2009) showed that when oxalate:Ca is higher than 1.0, dietary calcium availability is limited. The oxalate:Ca molar ratios of unprocessed and processed sorghum grains were below the critical level of 1.0. This implies that oxalate may not have adverse effects on bioavailability of dietary calcium in the studied samples.

The molar ratio of phytate*Ca:Zn ratio higher than 200 is reported to negatively influence the zinc bioavailability (Castro‐Alba et al., 2019). The ratios in both treated and untreated sorghum grain samples were within the recommended values and probably favor for bioavailability of zinc.

## 4 CONCLUSIONS

The study showed that premilling treatments had a significant (*p* < .05) impact on the nutritional compositions, antinutritional factors, and mineral bioavailability of sorghum flour. Among premilling treatments, malting significantly increased the minerals content but decreased concentration of antinutritional factors. This implies use of malted sorghum grains as compared to the raw sorghum grains significantly improved protein digestion and minerals bioavailability which contribute for better nutrition and health of the public in general and children under ages of five in particular. Therefore, malted sorghum‐based weaning food should be enhanced with combination of other nutrient‐dense food which could serve for infant and young children to support their early growth and development.

## CONFLICT OF INTEREST

The authors declare no conflict of interest.

## ETHICAL REVIEW

This study does not involve any human or animal testing.

## Data Availability

Data openly available in a public repository that issues datasets with DOIs.
